# TLR9 Agonist Protects Mice from Radiation-Induced Gastrointestinal Syndrome

**DOI:** 10.1371/journal.pone.0029357

**Published:** 2012-01-04

**Authors:** Subhrajit Saha, Payel Bhanja, Laibin Liu, Alan A. Alfieri, Dong Yu, Ekambar R. Kandimalla, Sudhir Agrawal, Chandan Guha

**Affiliations:** 1 Department of Radiation Oncology, Albert Einstein College of Medicine, The Montefiore Medical Center, Bronx, New York, United States of America; 2 Department of Pathology, Albert Einstein College of Medicine, The Montefiore Medical Center, Bronx, New York, United States of America; 3 Idera Pharmaceuticals, Inc., Cambridge, Massachusetts, United States of America; Roswell Park Cancer Institute, United States of America

## Abstract

**Purpose:**

Radiation-induced gastrointestinal syndrome (RIGS) is due to the clonogenic loss of crypt cells and villi depopulation, resulting in disruption of mucosal barrier, bacterial invasion, inflammation and sepsis. Intestinal macrophages could recognize invading bacterial DNA via TLR9 receptors and transmit regenerative signals to the neighboring crypt. We therefore investigated whether systemic administration of designer TLR9 agonist could ameliorate RIGS by activating TLR9.

**Methods and Materials:**

Male C57Bl6 mice were distributed in four experimental cohorts, whole body irradiation (WBI) (8.4–10.4 Gy), TLR9 agonist (1 mg/kg s.c.), 1 h pre- or post-WBI and TLR9 agonist+WBI+iMyd88 (pretreatment with inhibitory peptide against Myd88). Animals were observed for survival and intestine was harvested for histological analysis. BALB/c mice with CT26 colon tumors in abdominal wall were irradiated with 14 Gy single dose of whole abdominal irradiation (AIR) for tumor growth study.

**Results:**

Mice receiving pre-WBI TLR9 agonist demonstrated improvement of survival after 10.4 Gy (p<0.03), 9.4 Gy (p<0.008) and 8.4 Gy (p<0.002) of WBI, compared to untreated or iMyd88-treated controls. Post-WBI TLR9 agonist mitigates up to 8.4 Gy WBI (p<0.01). Histological analysis and xylose absorption test demonstrated significant structural and functional restitution of the intestine in WBI+TLR9 agonist cohorts. Although, AIR reduced tumor growth, all animals died within 12 days from RIGS. TLR9 agonist improved the survival of mice beyond 28 days post-AIR (p<0.008) with significant reduction of tumor growth (p<0.0001).

**Conclusions:**

TLR9 agonist treatment could serve both as a prophylactic or mitigating agent against acute radiation syndrome and also as an adjuvant therapy to increase the therapeutic ratio of abdominal Radiation Therapy for Gastro Intestinal malignancies.

## Introduction

Radiation-induced gastrointestinal syndrome (RIGS) remains one of the major limitations for delivering tumoricidal doses of abdominal radiation therapy (RT). It could also limit survival of victims in a mass casualty setting from nuclear accidents or terrorism. While supportive care with antibiotics, hydration and bone marrow transplantation can rescue radiation-induced bone marrow syndrome, currently there are no approved therapy for protecting or mitigating RIGS. Manifestations of RIGS are influenced by radiation dose with a short prodromal syndrome of nausea and vomiting upon exposure to whole body irradiation of 1.5 Gy. With higher doses (≥6 Gy), the prodromal syndrome is more marked, followed by a subacute syndrome of diarrhea and gastrointestinal bleeding in 2–5 days post-exposure. As doses reach over 8–10 Gy, the full-blown syndrome of RIGS with diarrhea, dehydration, sepsis and intestinal bleeding ensues with eventual mortality. In the clinic, higher doses from fractionated RT or large single fractions during sterotactic body radiation therapy (SBRT) can cause diarrhea and gastrointestinal bleeding, ulcer or fistula from breakdown of irradiated intestinal mucosa. The efficacy of cancer treatment by radiation and chemotherapeutic drugs is often limited by severe side effects that primarily affect the hematopoietic system and the epithelium of the gastrointestinal tract. Progress in understanding differences in the mechanisms involved in the responses of normal and tumor cells to genotoxic stress has led to the development of new rational approaches to selective protection of normal cells, such as suppression of apoptosis by pharmacological inhibition of p53 or activation of NF-k B. Another promising approach presented in this issue by Johnson et al. is based on the idea of using pharmacological inhibitors of cyclin-dependent kinases (CDKs) to convert normal cells into a radioresistant state by inducing reversible cell cycle arrest at the G1/S transition.

The pathophysiological mechanisms of RIGS is complex and involves loss of clonogenic crypt cells with eventual depopulation of the intestinal villi, defective regeneration of the irradiated intestinal stem cells and a systemic inflammatory response syndrome (SIRS) from a host of cytokines and growth factors released in the serum, following exposure to radiation and gut microbes [1], [2]. Survival from RIGS depends on the rate of the crypt depopulation and the efficiency and number of the residual clonogens, capable of regenerating crypt-villus units. Therefore, growth factors that can promote proliferation of intestinal crypt cells, such as, keratinocyte growth factor and interleukin-11 have been shown to be radioprotective for RIGS [3], [4], [5]. Similarly, pre-treatment with growth factors that inhibit cell cycle in regenerating crypts, such as, transforming growth factor-β1 (TGFβ1) and transforming growth factor-β3 (TGFβ3), promoted radioresistance in these cells. Besides the intestinal crypts where the putative intestinal stem cell (ISC) resides, the stroma or the ISC niche has also been postulated to be a target in RIGS. Thus, endothelial cells residing in the ISC niche, is particularly vulnerable to radiation. Growth factors, such as, basic fibroblast growth factor (bFGF) that prevented radiation-induced endothelial [6], [7], [8], [9], [10] cell death, also conferred radioprotection from RIGS.

Besides the endothelial cell the intestinal subepithelial immune effector cells, such as, gd-T cells and macrophages interact with the host enteric microbiota and modulate the intestinal regenerative response following radiation exposure [11]. Cellular signaling via the Toll-like receptor (TLR) and other pathogen-associated molecular pattern (PAMP) recognition molecules plays a critical role in the dynamic interactions between the host's enteric microbiota and innate immune system [12], [13]. Intestinal macrophages function as mobile “cellular transceivers” that “recognize” invading bacteria entering through the disrupted intestinal mucosa via the TLR receptors and transmit regenerative and repair signals to the neighboring intestinal epithelial progenitors in the ISC niche by forming immune synapses with the ISC [14]. Recently, a peptide derived from Salmonella Flagellin, which is a ligand for TLR5, was found to be radioprotective against RIGS in murine and primate models, possibly via NFκB activation [15]. TLR9 is present in the basolateral surface of crypt epithelial cells and could potentially sense bacterial invasion via TLR ligand binding. Earlier reports demonstrated that activation of TLR9 pathway by bacterial CpG-rich oligonucleotides could be beneficial in protecting against chemical models of intestinal injury [16]. TLR stimulation could also contribute to generation of anti-inflammatory signal and dampen intestinal sepsis [17]. We, therefore, hypothesized that systemic administration of TLR9 ligands could activate TLR signaling in the intestine and confer radioprotection and/or mitigation from RIGS. In this study we have used a novel TLR9 agonist containing synthetic immunomodulatory CpR (R = 2′-deoxy-7-dezaguanosine) dinucleotide and 3′-3′-attached novel structures that has been shown to induce potent TLR9-mediated immune responses [18], [19], [20], [21]. The presence of 3′-3′-attached structure provides higher metabolic stability and also optimal 5′-end presentation required for TLR9 recognition [18], [22], [23], [24]. These novel TLR9 agonists have been shown to induce potent Th1-type immune responses and a broad spectrum of antitumor activity in a number of tumor models [18], [25], [26], [27], [28]. A human selective TLR9 agonist, referred to as IMO-2055, is in phase II clinical trials for cancer [29]. Here we demonstrate that activation of the TLR9 pathway by a novel TLR9 agonist promoted intestinal crypt cell survival by inhibiting radiation-induced apoptosis and stimulated regeneration of the irradiated intestine, thereby, improving survival in a murine model of RIGS.

## Methods

### Animals

4–6 weeks old C57/Bl6 and BALB/c male mice were purchased from Jackson Laboratory (Portland, Main), and housed in the barrier facility at the Albert Einstein College of Medicine (Bronx, NY). Mice were maintained *ad libitum* and all studies were performed under the guidelines and protocols of the Institutional Animal Care and Use Committee of the Albert Einstein College of Medicine. The animal use protocol for this study was reviewed and approved by the Institutional Animal Care and Use Committee (IACUC) of Albert Einstein College of Medicine (IACUC approval# 20080703).

### Cell lines and maintenance

CT26 a mice colon carcinoma cell line (ATCC, Manassas, VA), IEC6 rat intestinal epithelial cell line (ATCC) and J774A.1 a mouse macrophage cell line (ATCC) were cultured according to ATCC recommended protocol. In brief CT26 was cultured in RPMI-1640 growth Medium and J774A.1, IEC6 were cultured in Dulbecco's Modified Eagle's Medium in presence of 10% fetal bovine serum. Cells were maintained in 37°C in presence of 5% CO2.

### TLR9 agonist Treatment

TLR9 agonist was synthesized, purified and characterized at Idera Pharmaceuticals as described previously [18], [30]. The purity of the final product was 95% with less than 0.1 EU/ml of endotoxin as determined by the Limulus assay (Bio-Whittaker Lonza Walkersville Inc., Walkersville, MD). TLR9 agonist was subcutaneously administered to C57Bl6 mouse (n = 15), 1 hour prior to or after whole body irradiation (WBI) at a dose of 1.0 mg/kg of body weight/mice. Control mice received subcutaneous injection of phosphate buffered saline (PBS) (Manassas, VA). In separate cohorts of mice (n = 15), we also inhibited the downstream signaling of TLR9 by administering a PBS-solubilized, MyD88 homodimerization inhibitory peptide, DRQIKIWFQNRRMKWKKRDVLPGT (Imegenex, San Diego, CA). Treatment with inhibitory peptide (0.5 mg/kg of body wt/alternate day) was for 21 days prior to irradiation and continued up to survival endpoint.

### Irradiation procedure

Animals were anesthetized by intraperitoneal injection of 100 µl of ketamine and xylazine (7∶1 mg/ml/mouse) and were exposed to whole-body irradiation (WBI) ^137^Cs γ-rays at a dose rate of 236 cGy/min (8.4–10.4 Gy). Abdominal irradiation (AIR) was performed using a 320 KvP, Phillips MGC-40 Orthovoltage irradiator at a 50 cm SSD with a 2 mm copper filter and a dose rate of 72 cGy/min. AIR was administered to the mice after shielding the thorax, head and neck and extremities and protecting a significant portion of the bone marrow, thus inducing predominantly RIGS.

### Histology

The intestine of each animal was dissected, washed in PBS to remove intestinal contents and the jejunum was fixed in 10% neutral buffered formalin prior to paraffin embedding. Tissue was routinely processed and cut into 5 µm sections for hematoxylin and eosin and immunohistochemical staining. All hemotoxylin and eosin (Fisher Scientific, Pittsburgh, PA) staining was performed at the Histology and Comparative Pathology Facility in the Albert Einstein Cancer Center.

### In vivo BrdU labeling and Proliferation rate assay

To visualize proliferation, each mouse was injected intraperitoneally with BrdU (120 mg/1 kg body weight, Sigma-Aldrich, St. Louis, MO), 2–4 hrs prior to sacrifice and tissue harvest. BrdU staining was performed on the sections. Briefly, 5 µm paraffin sections were prepared from the mid-jejunum. After paraffin removal and rehydration, tissue sections were incubated overnight at room temperature with a biotinylated monoclonal BrdU antibody (Zymed, San Francisco, CA). Streptavidin-peroxidase was used as a signal generator and diaminobenzidine (DAB) was used as a chromogen to stain BrdU-incorporated nuclei dark brown. Sections were counterstained with hematoxylin, dehydrated and mounted. Sections from mice not injected with BrdU were used as the negative control.

Crypts were identified histologically according to the criteria established by Potten C. S. [31]. Pictures of crypts were taken at high magnification Zeiss AxioHOME microscope and crypt epithelial cells (paneth and non-paneth) in mouse intestinal sections were examined using imageJ software and classified as BrdU positive if they had brown-stained nuclei from DAB staining or BRDU negative if they had blue stained nuclei. The Proliferation rate was calculated as a percentage of BRDU positive cells over the total number of cells in each crypt. A total of 30 crypts were examined per animal for all histological parameters. A regenerative crypt was confirmed by immunohistochemical detection of BrdU incorporation into five or more epithelial cells within each crypt, scored in a minimum of four cross-sections per mouse (n = 3/group).

### Determination of Crypt depth

Crypt depth was scored by taking pictures of crypts in H&E stained slides at 10× using a calibrated Zeiss AxioHOME microscope. The images were analyzed using imageJ software to measure the height in pixels from the bottom of the crypt to the crypt-villus junction. This measurement in pixels was converted to length in µM by with the following conversion factor: 1.46 pixels/µM.

### Detection of apoptosis in tissues in situ

To assess the number of apoptotic cells, in situ detection of cells with DNA strand breaks was performed in 4%-formalin-fixed, paraffin-embedded intestinal section by the TUNEL (TdT –mediated digoxigenin labeled dUTPnick and labeling) technique at Albert Einstein College of Medicine Dept. of pathology core facility. In brief, paraffin embedded section were de-paraffinized, rehydrated and stained using an ApopTag kit (Intregen Co, Norcross, Georgia). The Apoptosis rate in crypt cells was quantified by counting the percent of apoptotic cells in each crypt. Scoring was restricted to good longitudinal sections of the crypt in which the base of the crypt was aligned with all the other crypt bases, and evidence showed the crypt lumen to be present [32], [33].

### Immunohistochemistry to detect Macrophage

Pericryptal macrophages were stained by Alexa Fluor488-conjugated anti-mouse, F480 antibody (1∶50; Biolegend, San Diego, CA). Images were captured using a Zeiss SP2 confocal microscope at 40× optical zoom and the macrophages were counted by using the VolocitySoft Version 5.0 (Improvision, Waltham, MA) in 10 fields per mice in various cohorts (n = 3). Nucleus was stained with DAPI and pseudo colored with red.

### Xylose Absorption Assay

Functional regeneration of the irradiated intestines were determined by measuring intestinal absorption by a xylose uptake assay [34]. To quantify intestinal absorption as a physiological indicator of mucosal barrier integrity in different treatment group (n = 5/group) a xylose uptake assay was performed, at various time points (1, 3.5, 7 and 10 days) after irradiation. A 5% w/v solution of D-xylose (100 µl/mouse) in deionized water was administered orally by feeding tube. Animals were sacrificed after 2 hrs of D-xylose administration and blood samples were collected using heparinized blood collection tubes (BD Biosciences, San Jose, CA). Fifty µL of serum sample was added to 5 ml of phloroglucinol (1,3,5-trihydroxybenzene, Sigma Chemical Co., St. Louis, MO) color reagent (0.5 g of phloroglucinol, 100 ml glacial acetic acid and 10 ml of conc. HCL) heated to 100°C in a water bath for 4 min to allow optimum color development. After equilibration to room temperature, sample absorption was determined with the aid of a spectrophotometer set at a wavelength of 554 nm.

### In vitro clonogenic assay

Immediately after irradiation IEC6 cells were plated in 9.6 cm^2^ culture plate. The number of cells seeded depended on the radiation dose, so as to obtain a constant number of clones (50–100). The irradiated cells were allowed to grow until the surviving cells produced macroscopic colonies that could be readily counted, usually after 10–14 days following irradiation. Only colo- nies containing 50 or more cells were counted and the surviving fraction was calculated as follows: Surviving fraction = colonies counted/cell seeded×PE/100, where PE is the plating efficiency.

### Kaplan-Meier Survival Curve Analysis

The effect of irradiation and concomitant TLR9 agonist on mice survival/mortality was analyzed by Kaplan-Meier as a function of radiation dose using Sigma–Plot and Graphpad Prism-4.0 software for Apple PC.

## Results

### TLR9 agonist improves survival after WBI

**Figure 1:**
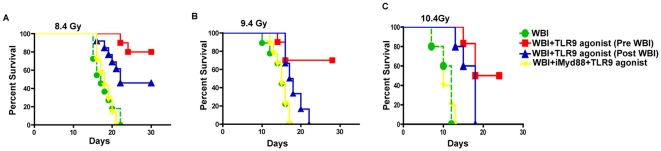
TLR9 agonist treatment protected C57Bl/6 mice from radiation-induced mortality. Kaplan-Meier survival analysis of C57Bl/6 mice treated with TLR9 agonist prior to WBI (A) 8.4 Gy; (B) 9.4 Gy and (C) 10.4 Gy. A significant survival was observed in TLR9 agonist-treated group, exposed to WBI of 10.4 Gy (p<0.03), 9.4 Gy (p<0.008), 8.4 Gy (p<0.002) (log rank test) compared to WBI alone or WBI+TLR9 agonist+iMyd88 cohorts. Treatment with TLR9 agonist after radiation exposure (WBI+Post-TLR9 agonist) had mitigation effect only at 8.4 Gy WBI. Administration of inhibitory peptide against Myd88 (iMyd88) completely inhibited radioprotective effect of TLR9 agonist.

RIGS presents with diarrhea, weight loss and death within 5–14 days in mice exposed to higher WBI doses [35]. We, therefore, administered escalating doses of single fraction WBI (8.4, 9.4 and 10.4 Gy) to C57Bl/6J mice to induce RIGS. Irradiated animals showed signs and symptoms of RIGS, including, diarrhea, black stools and weight loss. Mortality increased in control mice that received WBI and supportive treatment (antibiotics and PBS) with 100%, 80% and 0% of animals surviving 14 days, after 8.4, 9.4 and 10.4 Gy irradiation, respectively. Since TLR signaling modulates intestinal regeneration, we examined whether a TLR9-specific agonist, can protect against radiation lethality. Mice receiving TLR9 agonist, prior to WBI, had well-formed stools, maintained body weight (21.9±0.4 g in TLR9 agonist+WBI vs 15.18±0.4 g in WBI cohort, p<0.008) with a significant improvement in survival after 8.4 Gy (p<0.002), 9.4 Gy (p<0.008) and 10.4 Gy (p<0.03, log-rank test; Fig. 1) WBI. Mitigation effect of TLR9 agonist was only seen at 8.4 Gy WBI (p<0.01). In order to confirm the radioprotective role of TLR9 signaling, we inhibited a universal adapter protein, Myd88, which participates in TLR signaling by activating downstream NFkB. Intravenous injection of an inhibitory peptide that disrupts Myd88 homodimerization resulted in inhibition of radioprotection with eighty percent of mice dead within 14 days of TLR9 agonist+Myd88 inhibitior+10.4 Gy WBI, suggesting involvement of TLR9 signaling in mediating radioprotection (Fig. 1).

### TLR9 agonist reduces intestinal crypt cell apoptosis in irradiated mice

**Figure 2:**
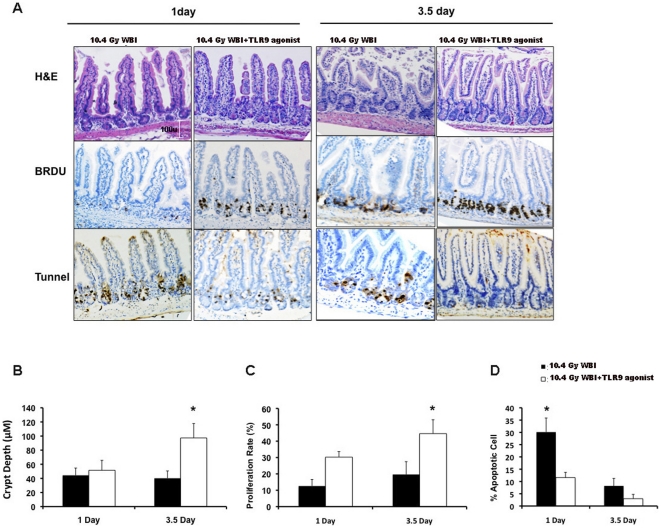
TLR9 agonist ameliorates RIGS. **A.** Representative (n = 3) histology from paraffin-embedded mid-jejunal sections of mice treated with TLR9 agonist+WBI versus WBI alone (20×). **Hematoxylin and Eosin staining** demonstrates larger crypt depth and elongated villi in the mid-jejunum of mice treated with TLR9 agonist and WBI, compared to mice treated with WBI. **BrdU immunohistochemistry** demonstrates increase in crypt cell proliferation after TLR9 agonist+WBI treatment, compared to WBI controls. Note an increase in BrdU-positive crypt cells in WBI-treated mice, which is further stimulated by TLR9 agonist treatment, 3.5 days after WBI. This is a physiological response of crypt survival after WBI. **TUNEL staining** demonstrates a decrease in TUNEL-positive, apoptotic cells in TLR9 agonist+WBI mice, when compared to WBI mice. **B–D.** **Bar graph of crypt depth, proliferation and apoptosis of crypt epithelial cells in mice treated with TLR9 agonist+WBI versus WBI alone.** Effect of TLR9 agonist on intestinal crypt depth (**B**), crypt proliferation rate (**C**) and tunnel positive apoptotic cells (**D**) at 1day and 3.5day post WBI. Mice treated with TLR9 agonist showed a significant resistance to radiation-induced apoptosis (p<0.001) at 1day post WBI with the increase in proliferation rate (p<0.002) and crypt depth at 3.5day post WBI (p<0.001), compared to irradiated control.

Since ionizing radiation induces apoptosis of intestinal crypt epithelial cells, we performed TUNEL assay to examine apoptosis of crypt epithelial cells, 1 and 3.5 day after WBI. There was a significant (p<0.001) decrease in the number of apoptotic nuclei in the jejunal crypts of TLR9 agonist+WBI-treated animals (11.6%±2.11) as compared with the irradiated controls (30.10%±5.74) (n = 3), suggesting that TLR9 agonist might increase the radio-resistance of the intestinal crypt compartment by inhibiting radiation-induced apoptosis (Figure 2 A&D).

### TLR9 agonist augments intestinal regeneration following irradiation

The characteristic tissue response to irradiation in intestine consists of apoptosis of ISCs and enterocytes, resulting in crypt depletion within day 1, followed by regeneration of surviving crypt clonogens, resulting in crypt microcolony formation at 3–4 days and eventual villi denudation by 7 days. We, therefore, examined the histopathology of irradiated intestines at days 1, 3.5 and 7 with hematoxylin-eosin staining and BrdU immunohistochemistry (n = 3). Compared to irradiated controls, the percentage of the BrdU-positive, crypt epithelial cells undergoing DNA synthesis was significantly (p<0.002) enhanced after TLR9 agonist treatment at 3.5 days post-WBI (Figs. 2A&C). This resulted in an increase in crypt depth and size (p<0.001), suggesting that TLR9 agonist treatment restitutes the crypt villous structures after radiation exposure (Figs. 2A&B).

### TLR9 agonist promotes recovery of intestinal absorption in irradiated mice

**Figure 3:**
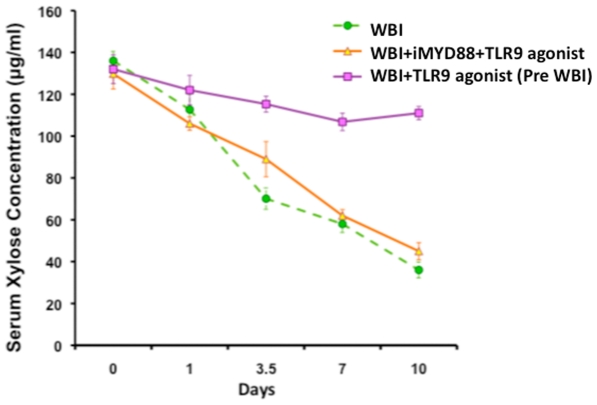
Xylose absorption assay. Mice treated with TLR9 agonist+WBI demonstrated significant recovery of xylose absorption post-WBI (p<0.0007 at 7 days), when compared to WBI controls. Animals treated with inhibitory Myd88 peptide, iMyd88 and WBI+TLR9 agonist failed to absorb xylose, indicating that TLR signaling was critical for the functional regeneration of intestine after radiation injury.

To evaluate the functional regeneration and absorptive capacity of the intestine, animals were fed xylose solution at various time points post-WBI. Since xylose is not metabolized in the body, serum xylose levels are a good indicator of the intestinal absorptive capacity. Two hours after a test feeding-dose, serum xylose levels were 130±4 µg/ml in non-irradiated mice. As expected, there was a progressive reduction in xylose absorption in irradiated mice with serum xylose level of 58±3.2 µg/ml in 7 days post-WBI. In contrast, mice treated with TLR9 agonist before WBI exhibited a significant recovery of xylose absorption 106.4±4.03 µg/ml (p<0.0007) at this time point. Xylose absorption continued to improve in the TLR9 agonist+WBI cohort up to 10 days post-WBI (Fig. 3), indicating quick restitution of the intestinal villi.

### TLR9 agonist enhances the recruitment of intestinal pericryptal macrophages

**Figure 4:**
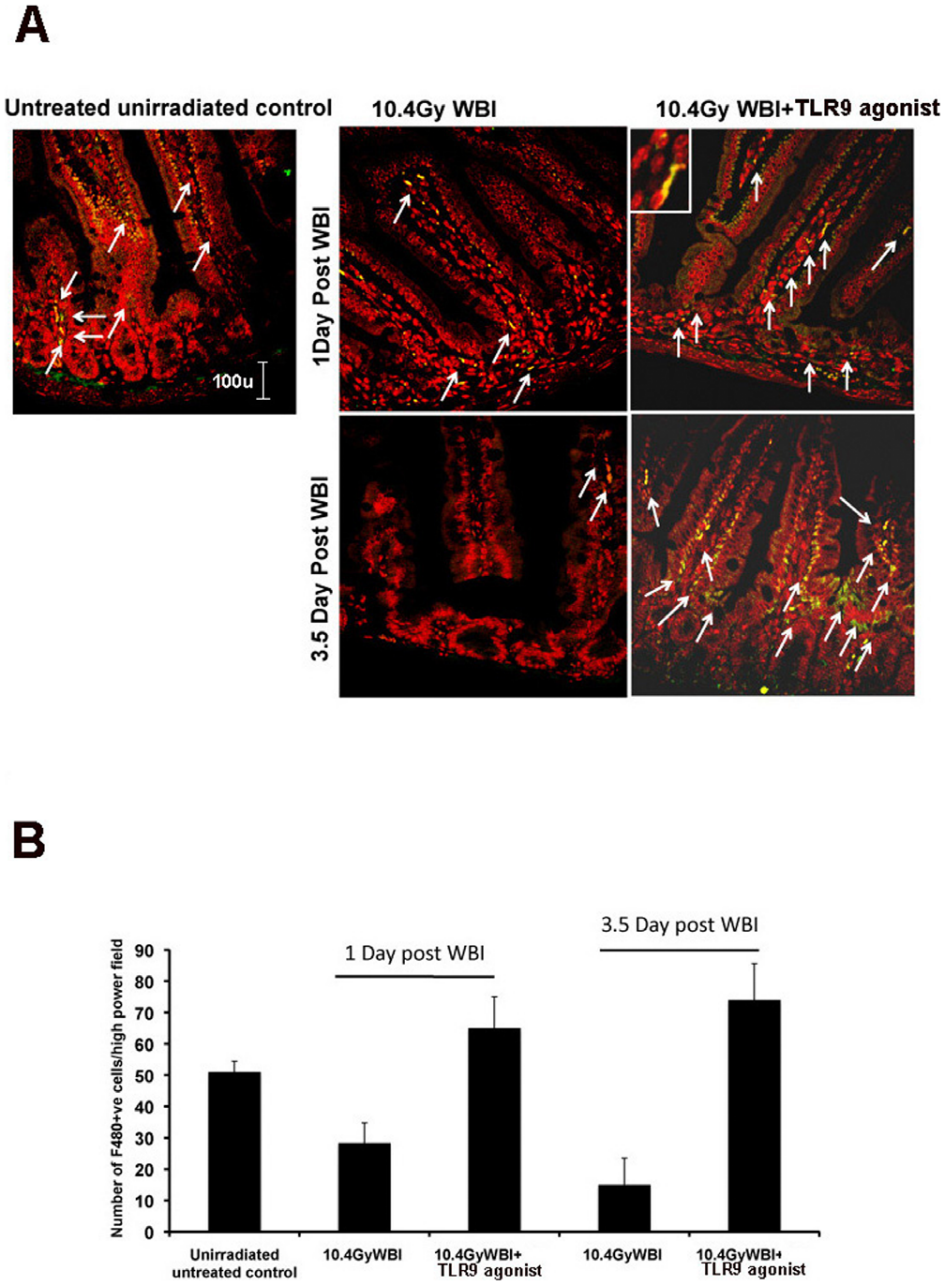
TLR9 agonist treatment increase the number of macrophages in pericryptal and lamina propria of intestine of mice treated with WBI. A. F480 Immunhistochemistry and confocal microscopic analysis and B. Quantification of number of intestinal macrophages. The number of F480+ve macrophages (indicated with arrow) increased at 1 day and 3.5 d post-WBI in the WBI+TLR9 agonist treatment group, compared to the WBI cohort. Nucleus was stained with DAPI (pseudo colored with red). Confocal microscopic images (40×) were magnified 2.3× (inset).

Intestinal pericryptal macrophages function as mobile “cellular transceivers” that coordinate inputs from luminal microbes and injured epithelium and transmit regenerative signals to neighboring stem cell and stem cell niche [14]. Moreover, activation of these macrophages was dependent on TLR activating signals coming from luminal microbiota. Immunohistological staining of jejunal cross-section showed a decrease in intestinal macrophage level in irradiated mice compared to non-irradiated control (p<0.009). However, TLR9 agonist treatment prior to WBI increased (p<0.006 at 1 day post WBI and p<0.001 at 3.5 day post WBI) F480+ve cells in lamina propria and pericryptal region suggesting a possible recruitment of intestinal pericryptal macrophages in WBI+TLR9 agonist-treated mice (Fig. 4).

### Transplantation of TLR9 agonist-treated macrophage improves survival of mice exposed to WBI

**Figure 5:**
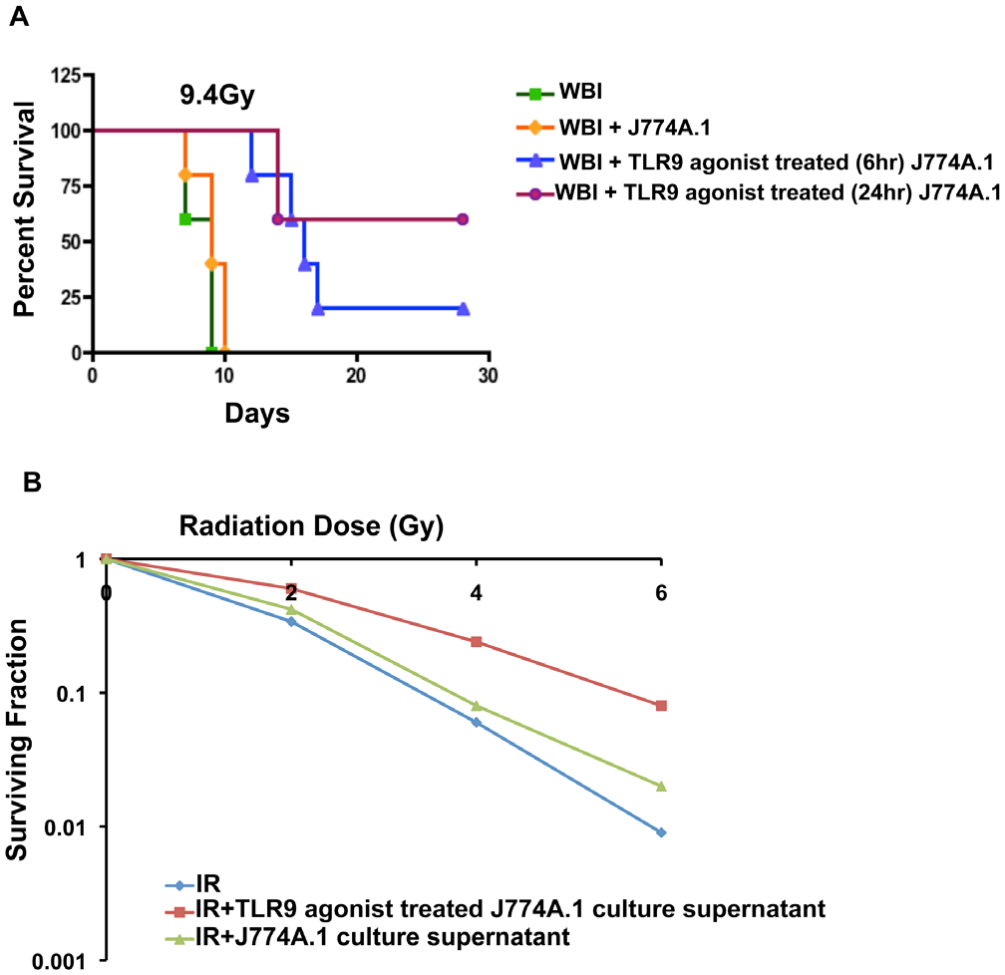
TLR9 agonist-activated macrophage mitigates RIGS. **A.** Macrophage cell line J774A.1 was incubated with TLR9 agonist in vitro for 24 hrs. BALB/c mice transplanted with syngeneic TLR9 agonist-activated J774A.1 macrophages, 1 hr after 9.4 Gy WBI, demonstrated significant improvement in survival, compared to WBI (p<0.002) or WBI+J774A.1 (p<0.005) cohorts. **B. Culture supernatant of TLR9 agonist-activated J774A.1 induces clonogenic survival of rat intestinal IEC6 following irradiation.** Incubation of IEC6 cells with culture supernatants from TLR9 agonist-activated J774A.1 cells followed by a clonogenic assay showed an increase in surviving fraction against incremental doses of radiation (2–6 Gy) compared to irradiated control.

We hypothesized that TLR9 agonist-mediated activation of TLR9 in intestinal pericryptal macrophages results in secretion of paracrine or trophic growth factors that induce crypt regeneration. To evaluate whether TLR9 agonist-activated macrophages were important to combat radiation damage, a macrophage cell line, J774A.1, was first stimulated in vitro with TLR9 agonist incubation for 6–24 hrs. Thereafter, TLR9 agonist-activated J774A.1 cells were transplanted by intravenous injection in syngeneic BALB/c (2×10^6^ cells/mice) mice, 1 hr after exposure to WBI (8.4–10.4 Gy). Transplantation of TLR9 agonist-activated J774A.1 cells rescued mice exposed upto 9.4 Gy WBI with significant improvement in survival compared to irradiated mice receiving either no cell transplantation (p<0.002) or J774A.1 cells that were not activated by TLR9 agonist (p<0.005) (n = 10) (Fig. 5A). This suggests that potential paracrine growth factors that are secreted by TLR9 agonist-treated J774A.1 cells might induce intestinal regeneration after irradiation. We confirmed this by incubating rat intestinal epithelial cells (IEC6) with culture supernatants from TLR9 agonist-treated J774A.1 cells following 2–6 Gy of irradiation in an in-vitro clonogenic assay and demonstrated an increase in clonogenic survival of irradiated IEC6 cells (survival fraction at 6 Gy 0.009±0.004 vs 0.08±0.01 p<0.0004) (Fig. 5B).

### TLR9 agonist increases the therapeutic ratio of abdominal irradiation

**Figure 6:**
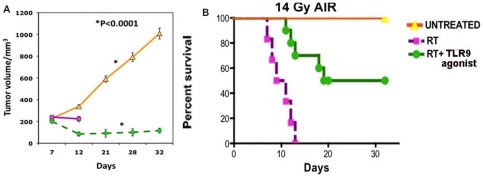
TLR9 agonist treatment improves the therapeutic ratio of whole abdominal RT for murine colorectal tumors. Effect of TLR9 agonist treatment on tumor growth rate of BALB/c mice (n = 5) irradiated with 14 Gy AIR. Significant delay in tumor growth (p<0.0001) was observed in AIR+TLR9 agonist groups (A) with improvement of survival of the mice (p<0.008) (B) compared to AIR alone and untreated mice.

Having demonstrated intestinal radioprotective effect of TLR9 agonist, we examined whether this effect by TLR9 agonist confers radioprotection to abdominal tumors. A murine colorectal, CT26 tumor cell line was injected subcutaneously under the abdominal wall of BALB/c mice. Mice with palpable, subcutaneous tumors were treated with TLR9 agonist, followed by whole abdominal irradiation (AIR) of 14 Gy [36]. AIR reduced the tumor growth but invariably produced 100% mortality of animals with a median survival time of 8±1.6 days. Compared to AIR alone, mice receiving TLR9 agonist 1 hr prior to AIR showed significant improvement in survival (median survival time 19±2 days vs 8±1.6 days). In AIR+TLR9 agonist treated group 50% of mice survived more than 30 days (Fig. 6B) and showed significant tumor growth retardation compared to untreated non-irradiated control (p<0.0001) (n = 10) (Fig. 6A). Comparison of the tumor volume upto day 12 post AIR (as 100% mice were died in AIR cohort by that day) showed significant difference between AIR and AIR+TLR9 agonist group (203±4.7 mm^3^ vs 85±3.8 mm^3^ p<0.003). Therefore these results demonstrates that administration of novel TLR9 agonist prior to irradiation may increase the radiosensitivity of the colorectal tumors while providing radioprotection to the normal tissues, including small intestine, thus demonstrating an increase in the therapeutic ratio of abdominal RT.

## Discussion

The gastrointestinal system is an early response organ to radiation. Rapid turnover of intestinal epithelial cells makes the intestinal mucosa especially vulnerable to high radiation exposure during RT or in nuclear accidents or terrorism. Thus, maintenance of intestinal homeostasis is very critical to combat against radiation-induced gastrointestinal injury. We have shown earlier that administration of an intestinal stem cell growth factor augment intestinal crypt cell proliferation in irradiated mice and improve survival by ameliorating RIGS [36]. In this report, we demonstrate that activation of TLR signaling by a synthetic ligand of TLR9 recruits pericryptal macrophages and provides growth stimuli to promote intestinal regeneration and inhibit apoptosis of crypt cells following radiation. TLR9 agonist treatment resulted in an increase in intestinal crypt depth and proliferative index and a decrease in crypt epithelial apoptosis with maintenance of the villi length following WBI. There was a marked and progressive restoration of the normal absorptive function of the intestine, as measured by xylose absorption test. TLR9 agonist-treated mice continued to survive beyond 4–6 weeks indicating that pretreatment with TLR9 agonist could ameliorate radiation-induced bone marrow syndrome in addition to RIGS. Besides radioprotection, TLR9 agonist treatment could mitigate RIGS to doses up to 8.4 Gy WBI. Finally, in a model of murine colorectal CT26 tumor, pretreatment with TLR9 agonist protected the mice from lethal RIGS, while improving the tumor suppression after whole abdominal RT. A possible cause of tumor radiosensitization could be potential immunomodulation, as demonstrated by Milas et al in murine colon, breast and lung cancer models [37].

Selective protection of normal tissue from radiation toxicity has also been achieved by modulating cell cycle arrest with different pharmacological agents [38]. Pharmacologic inhibitors of cyclin-dependent kinases (CDKs) induces reversible cell cycle arrest at the G1/S transition to convert normal cells into a transiently growth arrested, radioresistant state, where as tumor cells showed irreversible quiescence in response to these agents leading to induction of senscence or apoptosis. In separate studies, it has been shown that nutlin3, a P53 stabilizing agent, can be used in combination with mitotic inhibitors to induce reversible cell cycle arrest and thereby, cytoprotetion in normal cells, but not in P53 mutant tumor cells [39]. A differential stress resistance has also been observed between tumor cells and normal cells where short-term starvation or fasting induced chemoresistance in normal cells, while tumor cells remained sensitive to chemotoxicity [40]. It was postulated that mutated oncogenes prevented the switch to a protective stress response in tumor cells upon starvation for 24–48 hrs, while protecting normal cells across various model organisms, in humans, mice and yeast [40], [41]. Cancer cells are more resistant to apoptosis from acquiring mutations in genes, such as, p53 or inducing anti-apoptotic genes [42]. Upon genotoxic stress, normal cells with intact p53 either exhibit growth arrest or die by apoptosis, while tumor cells are indifferent to inhibition of apoptosis and are more prone to senescence [43]. Thus the anti-apoptotic role of TLR9 agonist could protect the crypt cells from radiation-induced apoptotic death but does not affect the colon cancer cells, which undergoes cell death or senescence after radiation exposure. TLR9-mediated immunomodulation might further enhance the therapeutic ratio of abdominal RT.

Our study suggests that commensal intestinal bacteria could play a significant role in maintaining intestinal homeostasis after radiation exposure. Activation of TLRs by commensal micobiota has been shown to augment mucosal restitution and modulate inflammatory response in sepsis in response to various forms of injury including radiation injury [44], [45], [46], necrotizing enterocolitis or experimental colitis [16], [47]. Pericryptal macrophages act as an important member of the ISC niche and crosstalks with intestinal subepithelial myofibroblast and ISC is essential to maintain intestinal epithelial homeostasis. These pericryptal macrophages recognize pathogen-associated molecular pattern (PAMP) signals from invading microbial organisms after radiation-induced mucosal injury. Various receptors, such as, TLR are critical for recognizing PAMP and activating macrophages to deliver regenerative signals to neighboring crypt ISCs [14]. Our results demonstrate that the universal TLR downstream adapter molecule Myd88 is involved in RIGS protection because inhibition of MyD88 homodimerization with an inhibitory peptide suppressed the radioprotective effects of TLR9 agonist. While, activation of NF-κB with the degradation of IκBα has been implicated as downstream pathway to TLR-Myd88 signaling in injury models, such as, dextran sulfate sodium (DSS)-induced experimental colitis [48], alternate pathways that involve inhibition of NF-κB have also been implicated in radioprotection [49].

Growth factors, such as, R-spondin1, KGF, TGFb and bFGF are known to protect the intestine from radiation or other cytotoxic injury by increasing the crypt cell proliferation and reducing apoptosis [5], [7], [35], [36]. Clonogenic survival of a rat intestinal epithelial cell, IEC6 was increased after incubation with culture supernatants of TLR9 agonist-activated macrophage cell line. Moreover, transplantation of TLR9 agonist-stimulated macrophages induced radio-mitigation against 9.4 Gy WBI, suggesting that TLR9 agonist-activated macrophages could secrete growth factors that might mediate the radioprotective effects of TLR9 agonist and TLR activation.

The vertebrate innate immune system has evolved to recognize highly conserved repeated molecular patterns in cell walls and nucleic acid structures of microbial pathogens using PAMP receptors, such as, TLRs. Of the various TLRs, TLR9 recognizes cytosine-phosphate-guanine (CpG) dinucleotides present in bacterial DNA and synthetic oligomers. Kandimalla et al synthesized designer TLR9 agonist containing two 5′ ends and synthetic purine bases, cytosine-phosphate-2′-deoxy-7-deazaguanosine dinucleotide motifs and demonstrated that these designer TLR9 agonists are potent ligands of TLR9 and are immunomodulatory in mouse, monkeys and humans [19]. The present report demonstrates that these TLR9 agonists could serve both as a prophylactic or mitigating agent against acute radiation syndrome and also as an adjuvant therapy to increase the therapeutic ratio in patients undergoing abdominal RT for GI malignancies.
